# Factors associated with experiencing sexual violence among female gender-based violence survivors in conflict-afflicted eastern Ukraine

**DOI:** 10.1186/s12889-021-10830-9

**Published:** 2021-04-24

**Authors:** Ariadna Capasso, Halyna Skipalska, Sally Guttmacher, Natalie G. Tikhonovsky, Peter Navario, Theresa P. Castillo

**Affiliations:** 1 NYU School of Global Public Health, New York University, 726 Broadway, New York, NY 10012 USA; 2 Ukrainian Foundation for Public Health, Kiev, Ukraine; 3 HealthRight International Ukraine Country Office, Kiev, Ukraine; 4 HealthRight International, New York, NY USA

**Keywords:** Sexual violence, Conflict, Humanitarian emergency, Ukraine, Non-domestic sexual violence, Intimate partner sexual violence, Gender-based violence, UN GBV-IMS

## Abstract

**Background:**

Since 2014, over 1.6 million people have been forcibly displaced by the conflict in eastern Ukraine. In 2014, 8% of reproductive-aged women in Ukraine had ever experienced sexual violence, compared to 5% in 2007. This increase was driven by non-domestic sexual violence. Our study examined characteristics of women in eastern Ukraine receiving psychosocial services following sexual violence compared to survivors of other forms of gender-based violence.

**Methods:**

Intake data collected between February 2016 and June 2017 by psychosocial service providers in five conflict-affected areas of Ukraine from women, aged 15–49, (*N* = 8525), was analyzed. Descriptive analysis and covariate adjusted logistic and negative binomial regressions were used to identify socioeconomic, incident and access to services factors associated with having experienced sexual violence compared to other forms of violence.

**Results:**

Among this sample of survivors receiving psychosocial services, 2.6% (*n* = 220) reported experiencing sexual violence. A majority of sexual violence acts reported were committed by non-domestic perpetrators (61.4%); followed by intimate partners (25.9%). Almost half of sexual violence cases occurred at home (49.1%). Experiencing sexual violence was positively associated with being younger, single and internally displaced, and negatively with engaging in unpaid labor, such as childcare. Women who experienced sexual violence delayed seeking care by 4 days compared to other gender-based violence survivors. Sexual violence survivors were less likely than physical violence survivors to have reported the incident prior to receiving care (adjusted odds ratio = 0.39; 95% confidence interval = 0.28–0.54).

**Conclusions:**

Non-domestic and intimate partner sexual violence were both prevalent in our sample. Compared to survivors disclosing other types of gender-based violence, sexual violence survivors appear to face unique barriers to reporting and accessing timely care. Prevention and outreach programs tailored to the specific vulnerabilities, such as displacement status, and needs of sexual violence survivors in conflict settings are urgently needed.

**Supplementary Information:**

The online version contains supplementary material available at 10.1186/s12889-021-10830-9.

## Background

### Sexual violence in humanitarian crises

Due to myriad factors, ranging from climate change to growing inequality, humanitarian crises have risen in recent decades, doubling in number between 2005 and 2017 [1]. These crises pose a direct threat to women’s right to health and to freedom from violence, rights enshrined in numerous international conventions [2]. Studies suggest that prevalence of all forms of gender-based violence (GBV), including sexual violence, is elevated in times of conflict or natural disaster [3–5]. It is estimated that more than 1 in 5 refugee and internally displaced women have disclosed ever experiencing sexual violence [6], although due to reporting barriers, the true prevalence is likely much higher [7]. Sexual violence not only represents a grave violation of women’s human rights, it also threatens their health and well-being, leading to an array of adverse physical, mental, sexual, and reproductive health outcomes [2, 8].

Humanitarian contexts may amplify the adverse psychological effects of sexual violence [9]. A small percentage of sexual violence survivors seek psychosocial or mental health services [10] and, among those who do, there are important delays in seeking care [11]. For example, among women seeking post-rape medical care, only 3.2% sought care within 72 h of the incident, and over one-third did so more than a year later [12]. Being younger and having the perpetrator be a family member are factors associated with delays in care [13].

### Sexual violence in conflict-affected Ukraine

Since 2014, over 1.6 million persons have been forcibly displaced in eastern Ukraine due to an ongoing military conflict, specifically in Luhansk and Donetsk [14]. An estimated 3.5 million people –both internally displaced persons (IDPs) and non-displaced local residents living in the conflict-affected regions– are in need of humanitarian assistance and protection in eastern Ukraine, an indication of the widespread deprivation caused by the conflict [14]. The humanitarian crisis has taken a significant, albeit uneven, toll on the Ukrainian economy, disproportionately impacting the livelihoods of IDP households [15].

The principal sources of information available on GBV in conflict-affected Ukraine are a UNFPA 2018 mixed-methods study and the 2019 Survey on Violence Against Women conducted by the Organization for Security and Co-operation in Europe (OSCE). The UNFPA study estimated that rates of GBV were three times greater among displaced women than among non-displaced women (15.2% vs. 5.3%) [15]. A majority (66%) of GBV survivors among non-displaced women knew their perpetrator personally. In contrast, proportionally more displaced GBV survivors experienced violence at the hands of strangers and of demobilized soldiers returning home. Study authors also pointed to the added risk for GBV occurring at entry-exit checkpoints along the contact line that separates government- from non-government-controlled areas (hereinafter ‘the contact line’), where hostilities are most intense [15].

Some evidence from humanitarian settings suggests that sexual violence by an intimate partner is more prevalent than by strangers [7]. The 2019 OSCE-led survey of women in Ukraine, which included women living in Donetsk and Luhansk, found the prevalence of intimate partner sexual violence (7%) to be higher than non-partner sexual violence (5%). Experience of physical and/or sexual violence was more prevalent among women whose partners had fought in an armed conflict compared to those whose partners were not combatants (31% vs. 15%) [16]. However, another national survey found a 3% increase of Ukrainian women who had ever experienced sexual violence from 2007 to 2014, 5 to 8%, respectively [17]. Whereas the percentage of women experiencing sexual violence from intimate partners and relatives remained unchanged over this period (3%), a greater share of sexual violence acts was perpetrated by strangers (4% in 2014 vs. 2% in 2007) [17].

In contrast to other conflict settings, the United Nations Office of the High Commissioner for Human Rights (OHCHR) found no evidence that sexual violence had been systematically used as a weapon of war in Ukraine [18]. However, entry-exit checkpoints along the contact line were identified as places of high vulnerability for sexual abuse against civilians. Furthermore, the report found that many cases of GBV were committed by military personnel, both near the contact line and in residential areas [18].

### GBV reporting and accessing services in conflict-affected Ukraine

The UNFPA survey found that displaced women in Ukraine tended to report GBV to law enforcement at lower rates than non-displaced women [15]. In this study, only 16% of displaced GBV survivors reported the incident to the police compared to 25% of local residents. Conversely, 5% of displaced and 3% of non-displaced GBV survivors disclosed their experience to healthcare professionals, including psychologists.

The overwhelming majority of GBV survivors (60%) chose to cope with their experience by confiding in family and friends; only 10% sought formal psychosocial support [16]. The UNFPA survey found marked differences in help-seeking attitudes between displaced and non-displaced women. Local residents were more likely than displaced women to express having no need for outside support (59% vs. 42%) and not being confident in the quality of the available local services (13% vs. 2%) [15]. Conversely, not knowing where to find help was the most cited reason given by displaced GBV survivors for not seeking services.

### Purpose of the study

There is a dearth of evidence regarding the characteristics of sexual violence experienced by women in conflict-affected areas of Ukraine. The vast majority of research on sexual violence in humanitarian settings has been conducted in sub-Saharan African countries [6, 7], with markedly different cultural, social and economic contexts. While recent surveys in Ukraine compared experiences of GBV by displacement status, they did not disaggregate incident, perpetrator and reporting characteristics by GBV type.

Using cross-sectional data collected from GBV survivors in care in eastern Ukraine, this study sought to identify patterns of sexual violence experienced by women living in areas of conflict. Among a sample of women receiving psychosocial support, this article examines: 1) the sociodemographic factors associated with experiencing sexual violence compared to other forms of GBV; 2) the characteristics of sexual violence perpetrators (e.g., age, relationship to survivor, occupation) and incidents (e.g., periodicity, location); 3) the time to access psychosocial support and institutional reporting of sexual violence incidents; and 4) whether these factors vary by residency status (local resident versus IDP).

Based on the existing literature, we hypothesize that: 1) internally displaced women will be more likely than non-displaced women to report experiencing sexual violence; 2) a disproportionate number of sexual violence perpetrators will be associated with combat operations (i.e., demobilized and active governmental and non-governmental soldiers) and a greater proportion of sexual violence incidents will occur at or near checkpoints; 3) incident characteristics (e.g., perpetrator and recurrence) will be different between women who experienced sexual violence compared to those experiencing other forms of GBV; and 4) compared to women experiencing other types of GBV, sexual violence survivors will delay seeking care and will be less likely to report the incident.

## Methods

### Design and sample

In November 2015, [masked] sought to expand access to mental health services for GBV survivors in eastern Ukraine through the deployment of mobile psychosocial support teams in five conflict-affected areas: Donetsk, Luhansk, Kharkiv, Dnipropetrovsk and Zaporizka. These teams, comprised of licensed psychologists and social workers, conducted outreach, provided direct services and documented cases of GBV utilizing an adapted UN-developed intake form, as described below. The mobile teams are integrated to local mental healthcare and social services networks through partnerships with local institutions, such as healthcare providers, police departments and social services, who refer women in need of counseling, either due to a discrete incident or to personal circumstances; in addition, the teams periodically organize information campaigns to make their services known to the community, resulting in an important number of self-referrals. Between February 2016 and June 2017, 26 mobile teams assisted 13,796 self-reported GBV survivors.

### Data collection

We analyzed data collected by mobile team service providers at intake using a standard form [19], which was developed by the UN Gender-Based Violence Information Management System (GBV-IMS) Global Team to collect harmonized GBV data in humanitarian settings and which has been evaluated in multiple contexts [20]. The form includes sociodemographic information, details about the incident and the perpetrator, past contact with relevant services and prior and future referrals. A group of humanitarian agencies, led by UNFPA, and which included HealthRight International and the Ukrainian Foundation for Public Health, translated and adapted the form to the Ukrainian context. Adaptation examples included modifying the form’s residency status, education, employment, and referral categories to make them relevant to the conflict and country setting (e.g., including options for army, police, and non-governmental soldier under perpetrator occupation). Mobile team service providers received a training session on the completion of the form.

### Procedures

Only data from clients who consented to releasing their de-identified information for research purposes were shared with our research team (*N* = 13,796) and of those 8525 were included in this analysis as per the eligibility criteria detailed below [19]. As secondary analysis of de-identified data, the New York University Institutional Review Board does not classify this study as human subjects research.

### Participants

**Fig. 1:**
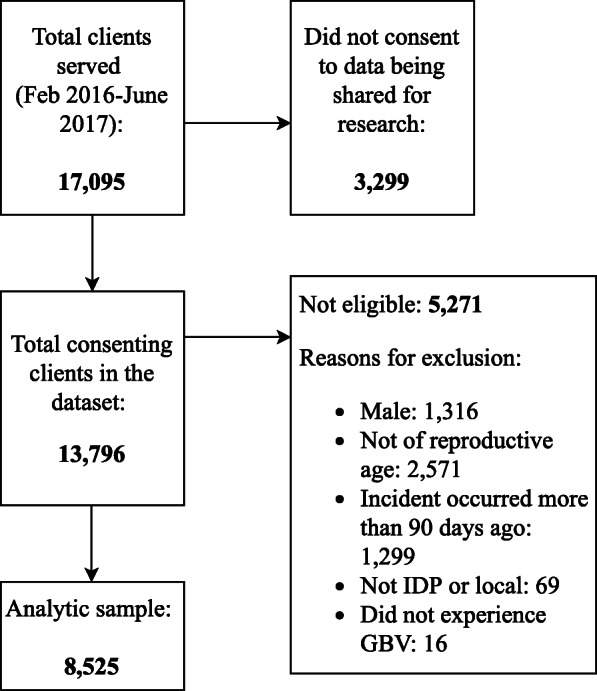
Flow diagram

We analyzed intake data from 8525 female GBV survivors receiving psychosocial services from mobile teams between February 2016 and June 2017. The final analytic sample includes all 15–49 year-old women who received psychosocial support for any of four forms of GBV (sexual violence, physical violence, economic violence or psychological abuse) within 3 months of the incident. We limited the analysis to women of reproductive age for purposes of comparability with the literature [21, 22] and because the vast majority of cases of sexual violence in our sample occurred within these ages. We excluded cases whose status was neither IPV nor local resident (*n* = 69) at the time the incident occurred and those classified as forced marriage because of low numbers (*n* = 8) as well as those classified as non-GBV, such as persons seeking services for psychological distress due to their financial situation or to having their homes shelled, because they were deemed outside the scope of the current study (Fig. 1 illustrates the Flow Diagram). The terms ‘outcome’ and ‘predictor’ variables below are used loosely to describe how the analysis was conducted and are not meant to indicate causality; given that the data is cross-sectional, only non-causal associations can be presented.

### Measures

#### Outcome variable

**Fig. 2:**
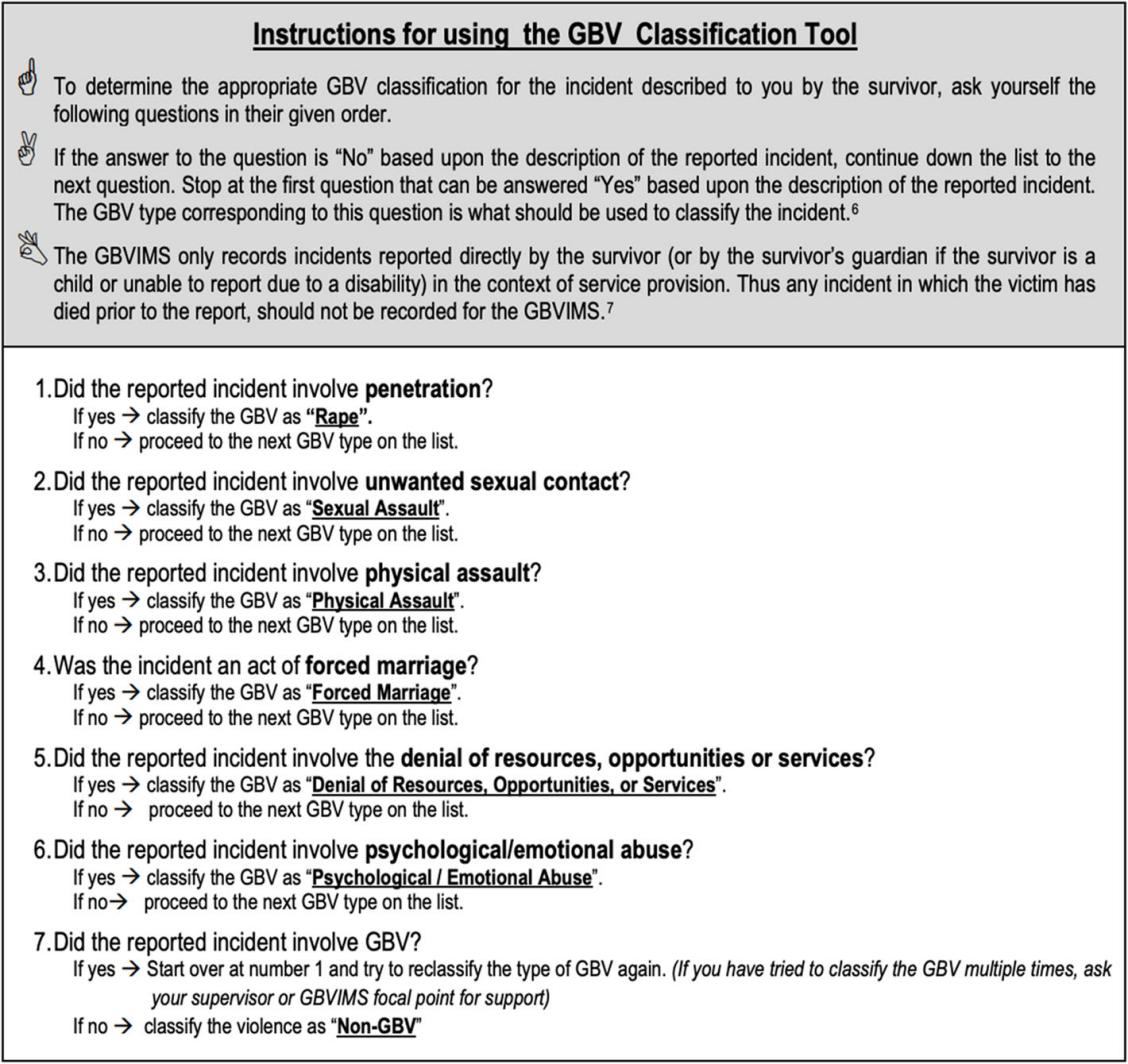
GBV Classification Instructions in the GBV-IMS. Source: UNFPA, UNHCR, & IRC. (2011). Chapter 4: The Intake and Consent Forms In G. G. Team (Ed.), *The Gender Based Violence Information Management System: User Guide.* Geneva: UNFPA, UNHCR & IRC

Determination of sexual violence was made by mobile team members using the GBV-IMS classification tool [19], which only captures one type of GBV per incident. The form instructs providers to select only one GBV type per case based on a series of questions asked in a specific order, as follows: 1) rape (if any type of penetration occurred); 2) sexual assault (if there was unwanted sexual contact); 3) physical assault (if there was physical battery); 4) forced marriage; 5) economic violence (in cases of denial of resources, opportunities or services; 6) psychological or emotional abuse (if the incident involved insults, name-calling, humiliation); and 7) no GBV (if none of the above). If, for example, the woman reported experiencing any unwanted sexual contact, the case would be classified as “sexual assault” and the provider moves on to the next section on the form. See Fig. 2 for instructions and categories and Additional file 1 for the full description of each GBV type. While this manner of classification facilitates standardization and consistency of data capture across multiple service providers, sites and countries, thus improving comparability, it comes at the cost of not capturing the multiple forms of GBV that women may have experienced [19]. We created a three-category variable for the descriptive analysis, as follows: sexual violence (includes rape and sexual assault); physical violence; and non-contact violence (includes economic and psychological violence). For multivariable analysis, we created two binary variables to compare survivors of sexual versus physical violence (0 = physical violence; 1 = sexual violence) and sexual versus non-contact violence (0 = non-contact violence; 1 = sexual violence) separately. Even though there are important differences among survivors of rape and sexual assault, these categories were combined because of low numbers (*n* = 28 rape and *n* = 192 of sexual assault incidents). Because of the GBV-IMS classification methodology, survivors who reported sexual violence may have also experienced physical and non-contact violence; and survivors who reported physical violence may have also experienced non-contact violence. We can, however, assert that non-contact violence survivors did not report sexual or physical violence, and that physical violence survivors did not report sexual violence. We reviewed the narrative description of all cases coded as non-GBV. Often, incidents of violence perpetrated by the intimate partner or a relative had been misclassified as non-GBV and described as “domestic violence – physical” or “domestic violence – psychological.” These cases were reclassified accordingly to their corresponding violence categories as follows: *n* = 1 as sexual violence; *n* = 117 as physical violence; *n* = 111 as economic violence; and *n* = 296 as psychological violence. In the majority of these cases, the perpetrator was the partner (*n* = 214) or a relative (*n* = 199).

#### Predictor variables

Sociodemographic variables were coded thus: Age at incident (continuous); status at time of incident (0 = non-displaced local residents; 1 = IDP); current marital status (0 = single or widowed; 1 = married or cohabitating; 2 = divorced or separated); number of children under 18 living with the client (0 = no children; 1 = 1+); and current employment (5 nominal categories). Age was skewed, therefore the median and interquartile range were reported. Age was maintained as a continuous variable to minimize information loss [23].

The incident description included two variables recoded as: recurrence (0 = assaulted once; 1 = assaulted multiple times or violence was ongoing); place of incident (0 = client or common home; 1 = perpetrator’s home; 2 = other indoor locations, including institutions such as healthcare clinics or social services buildings; 3 = outdoors). These categories reflect the most common places where sexual violence occurred.

#### Perpetrator characteristics included

Number of perpetrators (0 = 1; 1 = 2+; 2 = unknown); sex (0 = male; 1 = female or both); age of perpetrator in 4 age groups: under 26, 26–40, over 40, unknown); relationship to perpetrator (0 = intimate partner; 1 = family member; 2 = non-domestic, defined as perpetrators with no intimate or familial relationship to the survivor; 3 = unknown); perpetrator’s occupation (7 nominal categories, including governmental and non-governmental soldier); and demobilized soldier (0 = no; 1 = yes; 2 = unknown). Since there were no cases of sexual violence perpetrated by females and only two cases involved both males and females, the “female” and “both sexes” categories were combined. For age of perpetrator, the categories of 0–17 and 18–25, and 41–60 and 61+, were combined because of low numbers.

#### Access to services and reporting variables

1) Number of days from incident to intake. This variable was calculated by subtracting the date of the incident from the interview date. All women who accessed services more than 90 days following the incident were excluded from the analysis to improve comparability of the sample. 2) Whether the incident was reported was assessed with the question “Has the client reported this incident anywhere else?” (0 = no, 1 = yes) and if yes, the provider followed up with a question about where it was reported (4 nominal categories: 0 = health services; 1 = psychosocial services; 2 = police; 3 = other institutions). Multiple options were possible.

### Data analysis

We used Pearson’s chi-squared tests for categorical variables or Fisher’s exact test for categorical variables with any category with *n* ≤ 15 and two-sided Wilcoxon rank-sum tests for non-normally distributed continuous variables to evaluate whether sociodemographic and interpersonal factors and access to care differed among women who had experienced sexual violence, physical violence and non-contact violence [24], followed by post-hoc pairwise comparisons as needed. Because displacement may be associated with specific risk and protective factors, we also stratified the sample by residency status. A *p*-value below 0.05 was considered significant.

Using logistic regression models, we estimated unadjusted and adjusted odds ratios and 95% confidence intervals (CI) for associations between the predictor variables and sexual violence compared to physical and non-contact violence separately. In regression analyses results, we report sharpened False Discovery Rate (FDR) q-values in lieu of *p*-values to account for multiple hypothesis testing [25]. The first multiple model assessed differences in sociodemographic characteristics, adjusting for all sociodemographic variables. Applying blockwise entry, the subsequent models explored the association of perpetrator and incident characteristics, while adjusting for significant sociodemographic variables (age and marital and residency status). We used multiple logistic regressions to estimate the odds (and CIs) of having reported the incident among women who had experienced sexual violence compared to those who experienced physical and non-contact violence, separately. We used negative binomial regression to estimate days to seeking care, an overdispersed count variable. Variance inflation factors (VIF) were used to test the models for multicollinearity. Mean VIFs in the models ranged from 1.09 to 1.97, with the maximum individual VIF in any model being 4.59, below the 10 cutoff indicative of a multicollinearity problem [26]. To address the wide confidence intervals in some estimates, we conducted sensitivity analysis using penalized log likelihood estimation (‘firthlogit’ command). Because the results were similar to the initial analysis, we report solely the former for simplicity. All models used complete case analysis. Three predictor variables had missing data: number of children (missing < 1%), survivor’s occupation (missing < 1%), and violence recurrence (missing 3.1%). None of these variables had more than 5% of the values missing, thus unproblematic [27]. In the multivariable regression with the highest percentage of missing values, 4% of women had at least one missing value. All analyses were conducted in Stata 15.1 [28].

## Results

### Sociodemographic characteristics

Overall, 2.6% (*n* = 220) of the sample reported experiencing sexual violence. Of the sexual violence incidents reported, 12.7% involved rape and 87.3% other forms of sexual abuse. Displaced women were significantly more likely to report having experienced sexual violence than local women (3.1% vs. 2.1%). More than half (54.5%) of women who experienced sexual violence were single or widowed and slightly under half (49.1%) had no children, proportionately more than women who had not experienced sexual violence. Sexual violence survivors were, on average, 5 to 6 years younger than those not experiencing sexual violence. A disproportionate number of students (10.5%) and women who were not employed (29.6%) experienced sexual violence. Comparatively fewer women who engaged in unpaid labor (17.3%), such as child and elder care, experienced sexual violence compared to either physical or non-contact violence (Table 1).

**Table 1:** Descriptive Characteristics of 15–49-Year-Old Female GBV Survivors Seeking Psychosocial Support in Eastern Ukraine by Violence Type, *N* = 8525, 2016–2017

	Total	Sexual violence ^a^	Physical violence	Non-contact violence ^b^	*p*-value ^c^
	*n* (%)	*n* (%)	*n* (%)	*n* (%)	
	8525 (100.0)	220 (2.6)	2928 (34.3)	5377 (63.1)	
Age at incident [median (IQR)]	33 (28–39)	28 (22, 34)	34 (28, 39)	33 (28, 39)	< 0.001
IDP (vs. local) status at incident	2002 (23.5)	78 (35.5)	545 (18.6)	1379 (25.6)	0.001
Marital status					
Single or widowed	1818 (21.3)	120 (54.5)	478 (16.3)	1220 (22.7)	< 0.001
Married or Cohabitating	5242 (61.5)	68 (30.9)	2069 (70.7)	3105 (57.8)	
Divorced or Separated	1465 (17.1)	32 (14.6)	381 (13.0)	1052 (19.5)	
Number of children < 18					
No children	2324 (37.4)	108 (49.1)	778 (26.7)	1438 (26.9)	< 0.001
1+ child	6165 (72.6)	112 (50.9)	2140 (73.3)	3913 (73.1)	
Employment					
Professional	2025 (23.8)	56 (25.5)	654 (22.4)	1315 (24.5)	0.089
General labor	1814 (21.4)	38 (17.3)	659 (22.6)	1117 (20.8)	0.055
Unpaid labor	2714 (32.0)	38 (17.3)	909 (31.2)	1767 (33.0)	< 0.001
Not in the workforce	1709 (20.1)	65 (29.6)	621 (21.3)	1023 (19.1)	< 0.001
Student	233 (2.7)	23 (10.5)	71 (2.4)	139 (2.6)	< 0.001
Incident					
Recurrence of incident					
Once	5014 (60.7)	173 (79.0)	2197 (76.1)	2644 (51.3)	< 0.001
Multiple times	3244 (39.3)	46 (21.0)	690 (23.9)	2508 (4871)	
Place of incident					
Client or common home	6317 (74.1)	108 (49.1)	2369 (80.9)	3840 (71.4)	< 0.001
Perpetrator’s home	586 (6.9)	23 (10.5)	193 (6.6)	370 (6.9)	0.092
At an institution	793 (9.3)	49 (22.3)	109 (3.7)	635 (11.8)	< 0.001
Outdoors	829 (9.7)	40 (18.2)	257 (8.8)	532 (9.9)	< 0.001
Perpetrators					
Number of perpetrators					
1	7337 (86.1)	199 (90.5)	2666 (91.1)	4472 (83.2)	< 0.001
2+	790 (9.3)	12 (5.5)	110 (3.8)	668 (12.4)	< 0.001
Unknown	398 (4.7)	9 (4.1)	152 (5.2)	237 (4.4)	0.248
Sex of perpetrator					
Male	6896 (80.9)	218 (99.1)	2668 (91.1)	4010 (74.6)	< 0.001
Female/Both	1629 (19.1)	2 (0.9)	260 (8.9)	1367 (25.4)	
Age of perpetrator					
Under 26	690 (8.1)	32 (14.6)	171 (5.8)	487 (9.1)	< 0.001
26–40	4247 (49.8)	115 (52.3)	1672 (57.1)	2460 (45.8)	< 0.001
Over 40	3109 (36.5)	64 (29.1)	920 (31.4)	2125 (39.5)	< 0.001
Unknown	479 (5.6)	9 (4.1)	165 (5.6)	305 (5.7)	0.607
Relationship to perpetrator					
Intimate partner	5060 (59.4)	57 (25.9)	2165 (73.9)	2838 (52.8)	< 0.001
Family	1497 (17.6)	24 (10.9)	309 (10.6)	1164 (21.7)	< 0.001
No relation	1796 (21.1)	135 (61.4)	413 (14.1)	1248 (23.2)	< 0.001
Unknown	172 (2.0)	4 (1.8)	41 (1.4)	127 (2.4)	0.009
Perpetrator’s occupation					
Professional	1679 (19.7)	60 (27.3)	384 (13.1)	1235 (23.0)	< 0.001
General labor	2943 (34.5)	48 (21.8)	1238 (42.3)	1657 (30.8)	< 0.001
Not in labor force	1819 (21.3)	30 (13.6)	672 (23.0)	1117 (20.8)	0.001
Army or police	328 (3.9)	13 (5.9)	100 (3.4)	215 (4.0)	0.109
Non gov. soldier	218 (2.6)	0 (0.0)	146 (5.0)	72 (1.3)	< 0.001
Other	806 (9.5)	22 (10.0)	221 (7.6)	563 (10.5)	< 0.001
Unknown	732 (8.6)	47 (21.4)	167 (5.7)	518 (9.6)	< 0.001
Demobilized soldier					
No	7077 (83.0)	168 (76.4)	2330 (79.6)	4579 (85.2)	< 0.001
Yes	557 (6.5)	11 (5.0)	291 (9.9)	255 (4.7)	< 0.001
Unknown	891 (10.5)	41 (18.6)	307 (10.5)	543 (10.1)	< 0.001

*Note.* GBV Gender-based violence, *IDP* Internally displaced person, *IQR* Interquartile range, *NGO* Non-governmental organization

^a^ Includes rape and sexual assault. Because the GBV categories were mutually exclusive, sexual violence survivors may also have experienced other types of violence

^b^ Includes economic and psychological violence

^c^ *p*-value based on Pearson’s chi-square, Fisher’s exact or median test

### Incident and perpetrator characteristics

Nearly half (49.1%) of all reported incidents of sexual violence occurred at the women’s or common home, significantly fewer than the proportion of incidents of physical (80.9%) and non-contact violence reported (71.4%) happening at home (Table 1). A large proportion of sexual violence incidents occurred outdoors (18.2%) or at the perpetrator’s home (10.5%). No incidents of sexual violence were reported at IDP reception centers or at checkpoints along the contact line.

The overwhelming majority of sexual violence cases were by male (99.1%) perpetrators, acting alone (90.5%). Similar to other types of GBV, over half of sexual violence cases were committed by men between the ages of 26 and 40 (52.3%). However, proportionally more sexual violence acts were committed by men aged under 26 years, with 14.6% of sexual violence perpetrated by men under 26 compared to 5.8% of physical and 9.1% of non-contact violence cases. A majority (61.4%) of sexual violence acts were non-domestic; that is, perpetrated by men with no intimate or familial relationship to the survivor. In 21.4% of sexual violence cases (approximately 1 in every 5 cases), survivors did not know the perpetrator’s occupation or whether he was a demobilized soldier (18.6%), proportionally more than in cases of physical or non-contact violence (Table 1).

When stratifying the sample by residency status, intimate partners perpetrated proportionally more cases of sexual violence against local residents (32.9%) than against displaced women (14.5%); conversely, proportionally more cases of sexual violence against IDPs were non-domestic (67.1%) (Table S1 in the Supplementary material).

### Access to services and reporting

Table 2 contains information related to access to services and incident reporting. On average, women who experienced sexual violence took 4 days longer to access the services than those experiencing other types of violence. Only 26.4% of sexual violence survivors had reported the incident prior to coming to the mobile teams; this percentage was markedly lower than among those who experienced physical violence (50.7%) but comparable to those experiencing non-contact violence (28.7%). Of the 58 women who reported the sexual violence incident to any institution, 50.0% had reported it to the police; this is significantly lower than the proportion of physical violence survivors (69.2%) but higher than the proportion of non-contact violence survivors (31.8%) who filed a police report.

**Table 2:** Reporting Among 15–49-Year-Old Female GBV Survivors Seeking Psychosocial Support in Eastern Ukraine, N = 8525, 2016–2017

	Total	Sexual violence ^a^	Physical violence	Non-contact violence ^b^	*p*-value ^c^
	*n* (%)	*n* (%)	*n* (%)	*n* (%)	
	8525 (100.0)	220 (2.6)	2928 (34.3)	5377 (63.1)	
Days to access MT services [median (IQR)]	7 (2–17)	11 (5–23)	7 (2–17)	7 (3–17)	< 0.001
Previously reported incident	3086 (36.2)	58 (26.4)	1484 (50.7)	1544 (28.7)	< 0.001
If reported, where? ^d^					
Health services	285 (9.2)	4 (6.9)	184 (12.4)	97 (6.3)	< 0.001
Psychosocial services	1043 (33.8)	22 (37.9)	326 (22.0)	695 (45.0)	< 0.001
Police	1547 (50.1)	29 (50.0)	1027 (69.2)	491 (31.8)	< 0.001
Other institutions	723 (23.4)	11 (19.0)	200 (13.5)	512 (22.3)	< 0.001

*Note. GBV* Gender-based violence, *MT* Mobile teams, *IQR* Interquartile range

^a^ Includes rape and sexual assault. Because the GBV categories were mutually exclusive, women experiencing sexual violence may also have experienced other types of violence

^b^ Includes economic and psychological violence

^c^ *p*-value based on Pearson’s chi-square, Fisher’s exact or median test

^d^ Can report to multiple places

### Regression analysis: sociodemographic characteristics

Table 3 presents the results of the multivariable logistic regressions estimating the odds of experiencing sexual violence compared to physical violence; whereas Table 4 presents the odds of experiencing sexual violence compared to non-contact violence. When adjusting for sociodemographic characteristics, each additional year of age was associated with a 6% reduction in the odds of experiencing sexual violence (adjusted odds ratio [AOR] = 0.94; 95% confidence interval [CI] = 0.92–0.96) compared to physical violence and 5% compared to non-contact violence (AOR = 0.95; 95% CI = 0.93–0.97). Being internally displaced was associated with higher odds of experiencing sexual violence than either physical (AOR = 1.90; 95% CI = 1.39–2.59) or non-contact violence (AOR = 1.56; 95% CI = 1.16–2.08). Single and widowed women had higher odds of experiencing sexual violence compared to either physical (AOR = 4.28; 95% CI = 2.98–6.16) or non-contact violence (AOR = 2.67; 95% CI = 1.87–3.80) than married or cohabitating women. After adjustment, having children was of borderline significance and most of the employment categories were no longer significant. Only women who engaged in unpaid labor had lower odds of experiencing sexual violence compared to non-contact violence than professional women (AOR = 0.60; 95% CI = 0.38–0.93).

**Table 3:** Odds of Experiencing Sexual Violence Compared to Physical Violence Among 15–49-Year-Old Women Seeking Psychosocial Support in Eastern Ukraine, *N* = 3148, 2016–2017

	Crude Odds Ratios (OR)			Adjusted Odds Ratios (AOR)		
	OR	95% CI	*Sharpened q*-value	AOR	95% CI	*Sharpened q*-value
				Model 1: sociodemographic ^a^		
Age at incident	0.91	0.89, 0.93	0.001	0.94	0.92, 0.96	0.001
Residency status at incident						
Local resident	1.00			1.00		
IDP	2.4	1.79, 3.21	0.001	1.90	1.39, 2.59	0.001
Marital status						
Married or Cohabitating	1.00			1.00		
Single or widowed	7.64	5.58, 10.45	0.001	4.28	2.98, 6.16	0.001
Divorced or Separated	2.56	1.66, 3.94	0.001	2.59	1.66, 4.03	0.001
Number of children < 18						
No children	1.00			1.00		
1+ child	0.38	0.29, 0.50	0.001	0.71	0.50, 0.997	0.029
Employment						
Professional	1.00			1.00		
General labor	0.67	0.44, 1.03	0.020	0.86	0.55, 1.35	0.159
Unpaid labor	0.49	0.32, 0.75	0.001	0.68	0.43, 1.08	0.048
Not in the workforce	1.22	0.84, 1.78	0.079	1.16	0.77, 1.74	0.159
Student	3.78	2.20, 6.52	0.001	0.62	0.33, 1.17	0.048
				Model 2: incident ^b^		
Recurrence of incident						
Once	1.00			1.00		
Multiple times	0.85	0.60, 1.18	0.086	0.96	0.66, 1.37	0.200
Place of incident						
Client or common home	1.00			1.00		
Perpetrator’s home	2.61	1.63, 4.20	0.001	2.30	1.40, 3.79	0.002
At an institution	9.86	6.69, 14.54	0.001	5.48	3.58, 8.39	0.001
Outdoors	3.41	2.32, 5.02	0.001	2.00	1.32, 3.03	0.002
				Model 3: perpetrator ^b^		
Number of perpetrators						
1	1.00			1.00		
2+	1.46	0.79, 2.70	0.063	0.41	0.20, 0.85	0.013
Unknown	0.79	0.40, 1.58	0.102	0.36	0.15, 0.87	0.016
Sex of perpetrator						
Female/Both	1.00			1.00		
Male	10.62	2.62, 43.00	0.001	39.44	9.47, 164.20	0.001
Age of perpetrator						
Under 26	1.00			1.00		
26–40	0.37	0.24, 0.56	0.001	0.89	0.52, 1.52	0.175
Over 40	0.37	0.24, 0.59	0.001	0.94	0.53, 1.69	0.202
Unknown	0.29	0.13, 0.63	0.002	0.24	0.09, 0.62	0.003
Relationship to perpetrator						
Intimate partner	1.00			1.00		
Family member	2.95	1.80, 4.82	0.001	1.96	1.09, 3.51	0.017
No relation	12.42	8.95, 17.22	0.001	14.83	9.77, 22.51	0.001
Unknown	3.71	1.28, 10.69	0.006	29.23	6.79, 125.91	0.001

*Note: *OR* Odds ratio AOR* Adjusted odds ratio, *IDP* Internally displaced person

^a^ adjusted for all listed sociodemographic characteristics

^b^ adjusted for age and marital and residency status

**Table 4:** Odds of experiencing sexual violence compared to non-contact violence among 15–49-year-old women seeking psychosocial support in eastern Ukraine, *N* = 5597, 2016–2017

	Crude Odds Ratios (OR)			Adjusted Odds Ratios (AOR)		
	OR	95% CI	*Sharpened q*-value	AOR	95% CI	*Sharpened q*-value
				Model 1: sociodemographic ^a^		
Age at incident	0.92	0.91, 0.94	0.001	0.95	0.93, 0.97	0.001
Residency status at incident						
Local resident	1.00			1.00		
IDP	1.59	1.20, 2.11	0.001	1.56	1.16, 2.08	0.005
Marital status						
Married or Cohabitating	1.00			1.00		
Single or widowed	4.49	3.31, 6.09	0.001	2.67	1.87, 3.80	0.001
Divorced or Separated	1.39	0.91, 2.13	0.054	1.39	0.90, 2.14	0.079
Number of children under 18						
No children	1.00			1.00		
1+ child	0.38	0.29, 0.50	0.001	0.72	0.51, 0.999	0.038
Employment						
Professional	1.00			1.00		
General labor	0.80	0.53, 1.22	0.103	0.88	0.58, 1.35	0.222
Unpaid labor	0.50	0.33, 0.77	0.001	0.60	0.38, 0.93	0.021
Not in the workforce	1.49	1.03, 2.15	0.018	1.16	0.79, 1.71	0.185
Student	3.89	2.32, 6.51	0.001	0.91	0.50, 1.63	0.230
				Model 2: incident ^b^		
Recurrence of incident						
Once	1.00			1.00		
Multiple times	0.28	0.20, 0.39	0.001	0.30	0.22, 0.43	0.001
Place of incident						
Client or common home	1.00			1.00		
Perpetrator’s home	2.21	1.39, 3.51	0.001	1.83	1.13, 2.96	0.015
At an institution	2.74	1.94, 3.89	0.001	1.70	1.17, 2.45	0.008
Outdoors	2.67	1.84, 3.89	0.001	1.81	1.22, 2.69	0.005
				Model 3: perpetrator ^b^		
Number of perpetrators						
1	1.00			1.00		
2+	0.40	0.22, 0.73	0.002	0.3	0.15, 0.57	0.001
Unknown	0.85	0.43, 1.69	0.183	1.4	0.50, 3.88	0.212
Sex of perpetrator						
Female/Both	1.00			1.00		
Male	37.60	9.22, 149.72	0.001	66.34	16.28, 270.33	0.001
Age of perpetrator						
Under 26	1.00			1.00		
26–40	0.71	0.48, 1.01	0.049	1.11	0.70, 1.77	0.224
Over 40	0.46	0.30, 0.71	0.001	0.7	0.43, 1.16	0.084
Unknown	0.45	0.21, 0.95	0.019	0.22	0.08, 0.59	0.005
Relationship to perpetrator						
Intimate partner	1.00			1.00		
Family member	1.03	0.63, 1.66	0.200	1.85	1.08, 3.17	0.023
No relation	5.39	3.92, 7.39	0.001	10.01	6.88, 14.77	0.001
Unknown	1.57	0.56, 4.39	0.134	5.25	1.55, 17.84	0.01

*Note: *OR* Odds ratio AOR* Adjusted odds ratio, *IDP* Internally displaced person

^a^ adjusted for all listed sociodemographic characteristics

^b^ adjusted for age and marital and residency status

### Regression analysis: incident and perpetrator

When controlling for age and marital and residency status, GBV incidents against women were less likely to involve sexual violence at home. Compared to physical violence, GBV was more likely to be sexual when it took place at an institution (AOR = 5.48; 95% CI = 3.58–8.39) rather than at home (Table 3).

Odds of non-domestic sexual violence compared to physical violence were 14.83 (95% CI = 9.77–22.51) those of intimate partner violence. The same pattern held true when comparing odds of sexual violence to non-contact violence (AOR = 10.01; 95% CI = 6.88–14.77). In adjusted analysis, perpetrator age was no longer associated with sexual violence perpetration (Tables 3 and 4).

### Regression analysis: access to services and reporting

In adjusted analysis, women who experienced sexual violence were 61% less likely than those who experienced physical violence (AOR = 0.39; 95% CI = 0.28–0.54) to report the incident; but there were no differences in odds of reporting when compared to survivors of non-contact violence. Sexual violence survivors delayed seeking services compared to survivors of non-contact violence (incident rate ratio = 1.27; 95% CI = 1.08–1.49), but not to physical violence survivors (Table 5).

**Table 5:** Multivariable negative binomial and logistic regression models associated with service utilization among women receiving psychosocial care for GBV in eastern Ukraine, 2016–2017 ^a^

	IRR or AOR	95% CI	*Sharpened q*-value
Days to seek MT services (IRR)			
Sexual violence (vs. physical violence)	1.17	0.98, 1.40	0.115
Sexual violence (vs. non-contact violence)	1.27	1.08, 1.49	0.009
Previously reported incident (AOR)			
Sexual violence (vs. physical violence)	0.39	0.28, 0.54	0.001
Sexual violence (vs. non-contact violence)	0.85	0.63, 1.16	0.240

*Note*: *GBV* Gender based violence, *MT* Mobile team, *IRR* Incident rate ratio, *AOR* Adjusted odds ratio

^a^ Separate regressions were conducted to estimate access to services comparing survivors of sexual vs. physical and non-contact violence, separately, controlling for age and marital and residency status

## Discussion

In our sample of women receiving psychosocial support for GBV, sexual violence was the least common type of GBV reported. The 2.6% prevalence of sexual violence in our sample was lower than that reported in the UNFPA survey, which found that 5.6% of women who experienced any type of GBV experienced sexual violence [15]. However, this finding must be interpreted with caution. It is well known that survivors of sexual violence face multiple barriers to accessing psychosocial support, such as stigma, shame and perpetrator retaliation concerns [29]. A nationally-representative survey of women in Ukraine found that only about a quarter of women who ever experienced sexual and/or physical violence sought help, and among those who did, less than 2% sought psychosocial support [30]. Further, these barriers may be amplified by reduction in services, safety concerns and displacement in times of conflict [31].

Prior to embarking further in the discussion, we must revisit the data structure and remind the reader that: 1) all women in the study were GBV survivors; 2) women who reported experiencing sexual violence may have also experienced physical or non-contact violence; 3) women who reported experiencing physical violence also may have experienced non-contact-violence; 4) non-contact violence is the only category not affected by the mutually exclusive violence categorization used by the GBV-IMS; 5) GBV categories are based on self-disclosure and may be biased as a result of underreporting, particularly of sexual violence. In our sample, older age, being married and having children were associated with less likelihood of reporting sexual violence compared to the other types of GBV. Our findings are consistent with previous reports of an inverse association between age and GBV in general, which peaks in late adolescence and early adulthood [32], and sexual violence in particular [33]. A study among ever-married women in Ukraine, found that women aged 24 and under were at the highest risk for intimate partner sexual violence and that having children was a protective factor [33]. A novel finding from this study was that engaging in unpaid labor, such as elder and childcare, compared to being a professional, was associated with lower odds of reporting sexual violence compared to non-contact violence. Engaging in unpaid labor may have been a proxy for living with family and/or being part of a closer-knit social support network, perhaps conveying protection from sexual violence in the context of conflict-affected settings.

We found support for our first hypothesis that displaced women in our sample would report experiencing a greater share of sexual violence than non-displaced women. Research has found that social support is a protective factor for GBV while high population levels of stress and exposure to conflict are risk factors [32, 34]. One plausible hypothesis for this phenomenon is that displaced women may have been more exposed to sexual violence due to a breakdown of their social support networks, compounded by the stress of displacement within these networks. It is to be noted that all of the IDPs in our sample had resettled within the five regions that border the contact line, an area highly affected by the humanitarian impact of the conflict.

Evidence from population-based studies, regardless of the humanitarian context, suggests that unemployment, financial strain and household economic instability are associated with increased risk of GBV [32, 34, 35]. We found no evidence to support this association, finding no significant differences between unemployed and professional women in regard to likelihood of reporting sexual versus other types of violence. This study is only among survivors of GBV; economic strain may not be associated with experience of sexual violence in particular, compared to other types of violence, but to violence in general. Further, we did not ask about self or household income, which could be a better indicator of financial strain.

We could not make conclusions in regard to our second hypothesis that military involvement would be associated with sexual violence perpetration, due to the large number of unknowns in response to the perpetrator’s occupation. This is not surprising given that the majority of sexual violence survivors reported not knowing the perpetrator. We also did not find evidence to support the hypothesis that sexual violence occurred more often at IDP reception centers or at the checkpoints. Our findings differ from those of OHCHR, which identified numerous cases of conflict-related sexual violence at checkpoints and near the contact line [18]. However, the populations and samples are not comparable; neither is a representative sample of the population and the dates only partially overlap, with the OHCHR report spanning from March 2014 to January 2017 and ours from February 2016 to June 2017. In addition, underreporting is an issue. The same OHCHR report found that survivors who had to cross the contact line on an ongoing basis were afraid of reporting acts of violence due to fear of retaliation. It is possible that women who experienced sexual violence at checkpoints or the contact line either did not seek services from the mobile teams, misreported the location of the incident or did not consent to share their data for research purposes. Furthermore, the location of the mobile teams may have impeded some women from reaching them. During this time period, there were 26 operational mobile teams, which were unevenly distributed across cities and IDP camps within the five regions.

We found partial support for our third hypothesis that there would be marked differences in the characteristics of the incident by type of violence. In our sample of women receiving psychosocial support, a majority of the cases of sexual violence reported were isolated incidents perpetrated by young males acting alone, rather than ongoing intimate partner violence. Contrary to studies showing that, even in humanitarian settings, intimate partners are the most common perpetrators of sexual violence, the majority of cases of sexual violence in our sample were non-domestic (61.4%). After controlling for age, marital and residency status, odds of experiencing non-domestic sexual violence versus either physical or non-contact violence were higher than those by an intimate partner. Further, when stratifying by displacement status, we found that displaced women were less likely to experience intimate partner sexual violence and more likely to report non-domestic sexual violence than local women. The 2018 UNFPA survey found a similar pattern: non-domestic violence was proportionally much higher among displaced compared to non-displaced women [15]. A few factors might explain this pattern. Firstly, more displaced women may have been living separately from their husbands due to the conflict. Second, displaced women may have been reluctant to out their husbands as the perpetrator for fear of social and legal consequences (e.g., losing social benefits).

Nevertheless, we found that marital sexual violence was still an important problem. In more than one in four (25.9%) cases of sexual violence disclosed by women receiving services from the mobile teams, the perpetrator was the woman’s intimate partner. This is higher than the 16.4% reported in 2014 among Ukrainian women [30]. Even without direct exposure to war, the conflict might have led to societal changes in nearby regions that resulted in elevated levels of domestic violence, particularly among local women. Factors mentioned in the humanitarian literature include, financial strain, greater tolerance for violence, and greater numbers of demobilized soldiers who may have experienced war-related trauma [18, 34]. While Ukraine adopted legislation in 2017 to prevent domestic violence, it was only in 2019 that domestic violence was criminalized. Given documented stigma and normalization of intimate partner violence in Ukraine, it is possible that some women may not have been willing to report their partners as the perpetrator or might have been less willing to seek care [36]. In other settings, it has been found that survivors of intimate partner sexual violence often do not perceive the incident as a crime that requires seeking help [37–39].

In terms of access to services, we found partial support for our fourth hypothesis. Women who reported sexual violence took longer to access services, on average, and were less likely to have reported the incident previously than women who experienced other types of GBV. Sexual violence survivors in humanitarian contexts may face many barriers to accessing care and existing services are vastly underutilized [29]. Researchers attribute low service utilization to the stigma surrounding sexual violence, shame felt by survivors, and survivors’ concerns about confidentiality [40]. We found that about a quarter (26.4%) of sexual violence survivors had reported the incident previously, a rate comparable to non-contact violence survivors but markedly lower than that of physical violence survivors. Our findings are comparable to the 2014 Ukrainian survey that found that 27.9% of sexual violence survivors had sought some form of official support (psychological, legal, etc.) for the violence [30]. The same survey found that 33% of women who had received assistance found it futile [30], perhaps partially explaining why more survivors do not seek help. Of note, we found evidence of underreporting also among survivors of non-contact violence, highlighting the need to screen for and respond to the full spectrum of GBV when conducting research and developing policies and programs to facilitate GBV reporting and access to support services for survivors of violence.

### Limitations

Our study has several limitations that are important to consider. Firstly, as a cross-sectional data analysis, no causal inferences can be made with regards to the associations found. Second, since the study only included survivors of different forms of GBV who received psychosocial support, findings are not generalizable to the population as there most likely are systemic differences between survivors that do and that do not seek services, and we can make no assertions in regard to the prevalence of sexual violence in this region. Further, unmeasurable selection bias may have been introduced by restricting the analysis to data from participants who consented to sharing their information for research purposes, who likely also differ in systemic ways from those who did consent to sharing their information with the team.

The GBV-IMS intake form used for data collection was developed to inform programmatic decisions in humanitarian crises contexts. The form does not include psychological scales to measure mental health conditions that may influence health seeking behaviors and reporting, e.g., depression, trauma, etc. Furthermore, the form does not document if multiple forms of GBV occurred. Because of the order of the questions about GBV type in the form, there may be an unmeasured level of underreporting of physical and non-contact violence. In spite of this limitation, there would be no underreporting of sexual violence due to the form, since rape and sexual assault are the first two types of violence inquired about. However, there may be underreporting due to women being unwilling or unable to disclose to providers their experience of sexual violence.

Another limitation of this study is that data are self-reported. Particularly in the context of displacement, women may have been less likely to give details about the incident and perpetrators or to provide full information on their personal circumstances, perhaps for fear of losing benefits [41]. A national survey conducted soon after the start of the conflict revealed that 40.0% of women reported not being with a partner compared to 17.2% of women in Ukraine’s 2007 Demographic and Health Survey [17, 22]. This unwillingness to report family-level characteristics may be reflected in the elevated number of unknowns in our sample with respect to perpetrators’ occupations and military involvement.

The 95% confidence intervals for the estimates of perpetrator sex and relationship (category ‘unknown’) were wide because of low cell numbers. Estimates from penalized maximum likelihood logistic regression were similar to those of maximum likelihood estimation and the 95% confidence intervals remained wide (for example, for perpetrator sex: OR = 30.80; 95% CI = 8.54, 111.02). Thus, the estimates of perpetrator sex and relationship in the category ‘unknown’ lack precision; however, we can be fairly confident that there is a true significant positive association. We engaged in multiple hypothesis testing. However, the sharpened False Discovery Rate (FDR) q-values were largely consistent with the 95% confidence intervals, giving us confidence that we likely did not commit Type 1 Errors.

Despite these limitations, as one of the first analyses that specifically focuses on sexual violence in all the areas surrounding the ongoing conflict in Ukraine, this study contributes to an important evidence gap. In addition, another study strength is its large sample size, as well as the use of a validated assessment form, developed to document service provision in humanitarian emergencies and adapted to this population. The findings are an important contribution to the evidence on an understudied population and context, with recommendations that can be utilized to improve policies and programs for at risk women in this setting.

### Prevention, policy and research implications

Our findings are consistent with a generalization of GBV, in particular an increase of sexual marital violence in the context of armed conflict, and with different vulnerabilities to sexual violence faced by displaced versus local women. Interpersonal GBV is framed by power inequalities between men and women [42]. Conflict that leads to economic and social instability may result in increases in violence, including domestic violence, and reduce women’s ability to escape violent situations [43].

In terms of prevention implications, humanitarian response efforts should afford protections against sexual violence for younger, single women during conflict in the context of interactions with men outside of their family, including those involved in combat. Special prevention measures should also be put in place for women who experience forced displacement, whose increased dependency on non-partner males for basic sustenance in the context of limited social support may have placed them at increased risk for sexual violence.

It is worrisome that even among our sample of survivors accessing mental health care, many sexual violence survivors had not reported the incident before. Specialized outreach efforts are needed to reduce stigma and discrimination related to sexual violence and to reduce barriers to care for survivors of sexual violence.

Sexual violence by intimate partners is a stronger predictor of poor mental health outcomes than sexual violence by non-intimate partners [44, 45]. Given likely under-reporting of all sexual violence, and in particular violence by intimate partners, more programs are urgently needed to raise awareness, reporting and prevention of marital sexual violence in the population.

The data on sexual violence in conflict-affected Ukraine remains scarce. Additional quantitative and qualitative research is needed to better understand the circumstances in which sexual violence occurs; the barriers to care for survivors of sexual violence; the different factors affecting experience versus reporting of sexual violence; and to make recommendations for prevention, including research on the perpetrators of violence, and effective ways to provide support linkages for internally displaced women.

## Conclusions

This is one of the first analyses of patterns of sexual violence among women afflicted by the conflict in eastern Ukraine. In spite of the recent criminalization of domestic violence in Ukraine, urgent action is needed to prevent violence against women in the conflict-affected regions. More evidence is needed on effective, acceptable and feasible interventions for survivors of sexual violence in humanitarian settings [46]. Four GBV-prevention strategies may be feasible and scalable in such conflict-affected settings: to toughen the legal consequences for sexual violence offenders, including in cases of marital rape; to strengthen community engagement; to build awareness about resources available to survivors of sexual violence; and to increase the availability of anonymous and confidential services, ensuring due protection to the survivor [43]. Some of these mechanisms would be promising in Ukraine, with services tailored to women’s residency status, taking into consideration some of the different vulnerabilities identified in this study.

## Supplementary Information

**Additional file 1: Annex 1.** The six core types of GBV in the GBV-IMS. **Table S1.** Crosstabs of violence type by relationship to perpetrator, stratified by residency status.

## Data Availability

The datasets used and/or analyzed during the current study are available from the corresponding author on reasonable request.

## Abbreviations

AOR: Adjusted odds ratio
CI: Confidence interval
GBV: Gender-based violence
GBV-IMS: Gender-Based Violence Information Management System
IDP: Internally displaced person
OSCE: Organization for Security and Co-operation in Europe
OHCHR: Office of the United Nations High Commissioner for Human Rights
UN: United Nations
UNFPA: United Nations Population Fund
VIF: Variance inflation factor
