# Supplementary material for: Factors associated with experiencing sexual violence among female gender-based violence survivors in conflict-afflicted eastern Ukraine
Source: BMC Public Health. 2021 Apr 24;21:789. doi: 10.1186/s12889-021-10830-9 (PMC8067375; doi:10.1186/s12889-021-10830-9)
Supplement: Supplementary file 1 — Additional file 1: Annex 1. The six core types of GBV in the GBV-IMS. Table S1. Crosstabs of violence type by relationship to perpetrator, stratified by residency status. [file 12889_2021_10830_MOESM1_ESM.docx]

**Supplementary material**

Annex 1. **The six core types of GBV in the GBVIMS.**

Below is the definition of the six core types of GBV as defined in Annex B of the GBVIMS Classification Tool (available at <http://gbvims.com/wp/wp-content/uploads/ClassificationTool_Feb20112.pdf>, Accessed July 7, 2020).

1. Rape: non-consensual penetration (however slight) of the vagina, anus or mouth with a penis or other body part. Also includes penetration of the vagina or anus with an object.
2. Sexual Assault: any form of non-consensual sexual contact that does not result in or include penetration. Examples include: attempted rape, as well as unwanted kissing, fondling, or touching of genitalia and buttocks. FGM/C is an act of violence that impacts sexual organs, and as such should be classified as sexual assault. This incident type does not include rape, i.e., where penetration has occurred.
3. Physical Assault: an act of physical violence that is not sexual in nature. Examples include: hitting, slapping, choking, cutting, shoving, burning, shooting or use of any weapons, acid attacks or any other act that results in pain, discomfort or injury. This incident type does not include FGM/C.
4. Forced Marriage: the marriage of an individual against her or his will.
5. Denial of Resources, Opportunities or Services: denial of rightful access to economic resources/assets or livelihood opportunities, education, health or other social services. Examples include a widow prevented from receiving an inheritance, earnings forcibly taken by an intimate partner or family member, a woman prevented from using contraceptives, a girl prevented from attending school, etc. Reports of general poverty should not be recorded.
6. Psychological / Emotional Abuse: infliction of mental or emotional pain or injury. Examples include: threats of physical or sexual violence, intimidation, humiliation, forced isolation, stalking, harassment, unwanted attention, remarks, gestures or written words of a sexual and/or menacing nature, destruction of cherished things, etc.

| Table S1  Crosstabs of violence type by relationship to perpetrator, stratified by residency status | | | | | | | | | | | |
| --- | --- | --- | --- | --- | --- | --- | --- | --- | --- | --- | --- |
|  |  | Total | Sexual violence | Physical violence | Non-contact violence |  | Total | Sexual violence | Physical violence | Non-contact violence |  |
|  |  | n (%) | n (%) | n (%) | n (%) | *p*-value | n (%) | n (%) | n (%) | n (%) | *p*-value |
|  |  | Local resident | | | | | IDP | | | | |
| Relationship to perpetrator | |  |  |  |  |  |  |  |  |  |  |
|  | Intimate partner | 4216 (65.1) | 46 (32.9) | 1884 (79.3) | 2286 (57.8) | <0.001 | 844 (44.9) | 11 (14.5) | 281 (55.1) | 552 (42.6) | <0.001 |
|  | Family | 1212 (18.7) | 10 (7.1) | 274 (11.5) | 928 (23.5) | <0.001 | 285 (15.2) | 14 (18.4) | 35 (6.9) | 236 (18.2) | <0.001 |
|  | No relation | 1044 (16.1) | 84 (60.0) | 219 (9.2) | 741 (18.7) | <0.001 | 752 (40.0) | 51 (67.1) | 194 (38.0) | 507 (39.2) | <0.001 |
| *Note.* *p*-value based on Fisher's exact tests IDP = internally displaced person | | | | | |  |  |  |  |  |  |
